# Meaningful Partnerships: Stages of Development of a Patient and Family Advisory Council at a Family Medicine Residency Clinic

**DOI:** 10.2196/12105

**Published:** 2019-03-20

**Authors:** Jeffrey D Schlaudecker, Keesha Goodnow, Anna Goroncy, Reid Hartmann, Saundra Regan, Megan Rich, Adam Butler, Christopher White

**Affiliations:** 1 Research Division Department of Family and Community Medicine University of Cincinnati Cincinnati, OH United States; 2 The Christ Hospital/University of Cincinnati Family Medicine Residency Program Department of Family and Community Medicine University of Cincinnati Cincinnati, OH United States

**Keywords:** advisory committee, patient-centered care, patient engagement, graduate medical education

## Abstract

**Background:**

Partnering with patients and families is a crucial step in optimizing health. A patient and family advisory council (PFAC) is a group of patients and family members working together collaboratively with providers and staff to improve health care.

**Objective:**

This study aimed to describe the creation of a PFAC within a family medicine residency clinic. To understand the successful development of a PFAC, challenges, potential barriers, and positive outcomes of a meaningful partnership will be reported.

**Methods:**

The stages of PFAC development include leadership team formation and initial training, PFAC member recruitment, and meeting launch. Following a description of each stage, outcomes are outlined and lessons learned are discussed. PFAC members completed an open-ended survey and participated in a focus group interview at the completion of the first year. Interviewees provided feedback regarding (1) favorite aspects or experiences, (2) PFAC impact on a family medicine clinic, and (3) future projects to improve care. Common themes will be presented.

**Results:**

The composition of the PFAC consisted of 18 advisors, including 8 patient and family advisors, 4 staff advisors, 4 resident physician advisors, and 2 faculty physician advisors. The average meeting attendance was 12 members over 11 meetings in the span of the first year. A total of 13 out of 13 (100%) surveyed participants were satisfied with their experience serving on the PFAC.

**Conclusions:**

PFACs provide a platform for patient engagement and an opportunity to drive home key concepts around collaboration within a residency training program. A framework for the creation of a PFAC, along with lessons learned, can be utilized to advise other residency programs in developing and evaluating meaningful PFACs.

## Introduction

### Background

The core concepts of patient- and family-centered care (PFCC) are based on meaningful partnerships between patients, families, and the health care team. These partnerships promote dignity and respect; encourage information sharing between the provider and patient; foster participation in shared decision making; and cultivate collaboration between the patient, family, and health care team involved in the patient’s care [1]. Collaboration and participation across all ages and locations of care ensure that patients and their families act as included partners in care. Partnering with an active and engaged patient and family generates improved patient satisfaction and delivers upon the goal of higher quality care [2,3]. Providing the authentic voice of patients and families with the health care team is essential in moving health care to a fuller realization of optimized health for all. A patient and family advisory council (PFAC) is a group of patients and family members working together collaboratively with providers and staff to improve health care. A PFAC has shown success in several outcomes including patient experience and satisfaction [4,5]. The Centers for Medicare and Medicaid Services (CMS) recently introduced an innovation in primary care transformation, the Comprehensive Primary Care Initiative Plus (CPC+) [6]. Of the tenets of CPC+, 1 is patient and caregiver engagement, with the establishment of a PFAC being one requirement to fulfill that tenet [7,8].

The Christ Hospital Family Medicine Center is located in Cincinnati, Ohio. A total of 9 faculty providers and 26 resident physicians provide full-spectrum primary care for approximately 7000 patients, with 23,000 annual visits in a practice that is 60% Medicare/Medicaid and the remainder private insurance. The office was certified as a patient-centered medical home in 2010.

### Objectives

This study describes 1 program’s experience launching a PFAC within a family medicine residency clinic. We discuss the stages of PFAC development, which include PFAC leadership team formation and initial training, member recruitment, and meeting launch. PFAC evaluation included an open-ended survey and focus group interview at the completion of the first year.

## Methods

### Patient and Family Advisory Council Leadership Team Formation and Initial Training

A desire for family medicine resident physicians to engage with patients in office-based practice improvement initiatives was broadcast to faculty stakeholders, including the program director and clinic medical director. These faculty stakeholders agreed that a PFAC could be an ideal educational tool at our residency program. Requirements of the CMS’s 2016 CPC+ program of primary care payment restructuring to include a PFAC additionally piqued the interest of the health system and clinic medical director. A project manager was hired to manage the day-to-day operations of a related grant, and this person additionally served as the PFAC coordinator. Following approval of the residency program director and clinic medical director, the PFAC leadership team was formed. This team included several authors of this paper, including 2 residency faculty members and the PFAC coordinator.

Initial steps of the PFAC leadership team focused on identifying key practice stakeholders including the clinic nurse manager. Exploratory meetings were held with clinic and residency leadership to bolster agreement on the goal of the PFAC. The PFAC would be utilized to introduce patient voice into clinic issues, including residency-based clinic quality improvement projects. In addition, our PFAC would serve as a training model for residents to learn about the benefits of meaningful collaboration in their future practices.

To attain knowledge about the development and implementation of a PFAC, the PFAC leadership team attended a national 3-day seminar hosted by The Institute for Patient and Family Centered Care [1]. The seminar was a platform to learn from experts fostering genuine partnerships with patients, family members, and health care teams to improve the safety and quality in health care. Following this initial training, the team completed a formal action plan addressing the next steps needed to recruit and launch a PFAC.

### Patient and Family Advisory Council Member Recruitment

On the basis of best-practice examples, it is critical to identify a representative sample of patients and family members who have a wide variety of clinic experiences and reflect the diversity of the patient population. It is not necessary for all advisors to have universally positive experiences with the health care system or clinic; however, all PFAC members should have a desire to see the clinic improve. Outreach methods to enlist advisors included brochures and posters. A pocket information card we developed was our most effective recruitment strategy. Our providers carried this business card-sized tool in the clinic, which allowed them to invite engaged patients or family members in real time to consider joining our PFAC during the clinic visit.

Once patient or family members were identified as potential PFAC members, the medical director would call prospective candidates to personally invite them and validate the partnership between provider and patient. This point of contact also served to obtain permission for the PFAC coordinator, who was not an employee of the organization owning the clinic, to call to further discuss the PFAC. The PFAC coordinator then called each potential PFAC member to provide detailed information and conducted a short phone interview. The aim of this phone call was to communicate the purpose of the PFAC, describe the role of advisors, set expectations for involvement, and query potential barriers to attending meetings. In addition, patients and family members shared personal information and experiences that were helpful in guiding PFAC design. During the phone interview, the PFAC coordinator also obtained motivation and rationale for involvement in the PFAC. Although we have not barred a patient or family member from membership, we recognize that having an awareness of personal agendas is important to ensure that there is no conflict between patient or family member motivation and PFAC goals. At the completion of the call, an application was sent to potential PFAC members to collect contact information and preferences regarding meeting time.

**Figure 1:**
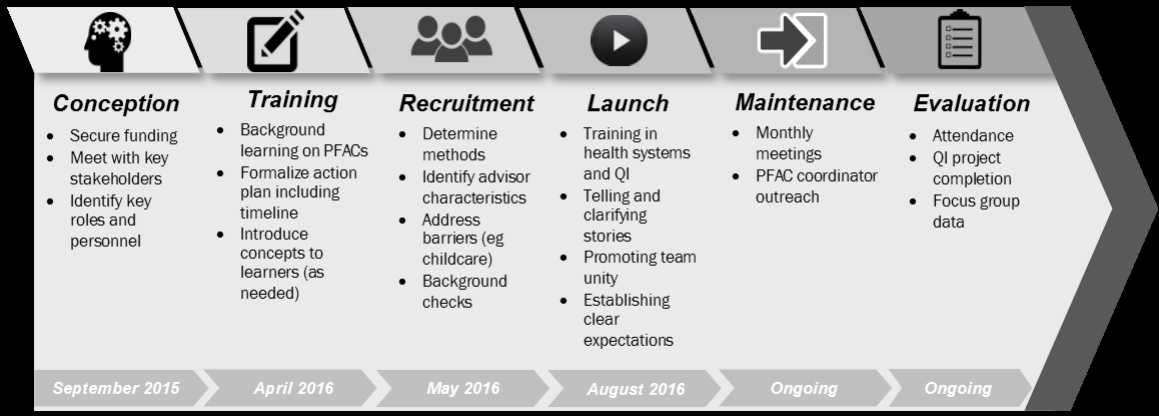
Patient and family advisory council creation timeline. PFAC: patient and family advisory council; QI: quality improvement.

Our PFAC membership included staff and physician members as well as patients and family members. Resident participation as PFAC members was solicited via email; 4 residents expressed interest and enthusiasm for participating. To maximize attendance, the PFAC leadership team decided on a monthly meeting schedule held from 5:00 pm to 6:00 pm on Wednesday evenings. The PFAC leadership team developed a curriculum for introducing health care–related topics, such as Health Insurance Portability and Accountability Act and quality improvement strategies, to the new PFAC members. A timeline for the process is depicted in Figure 1.

In accordance with hospital requirements, all volunteers, including PFAC advisors, completed a background check. As advisors occasionally encounter other patients and family members while attending meetings, it was important to follow this protocol to protect patient safety and privacy. Each PFAC member was asked to commit to a 1-year term with the option to re-enroll at the end of the term. This was critical in building a sense of community and trust for the partnership to be effective, while also providing a time-limited commitment that acknowledges busy lives. To support the advisors in keeping this commitment, free childcare was provided on site. Neither office staff nor PFAC advisors were reimbursed for their time, although snacks were provided at every meeting. The final composition of the PFAC was 18 PFAC members, including 8 patient/family advisors, 4 staff advisors, 4 resident advisors, and 2 faculty advisors.

**Figure 2:**
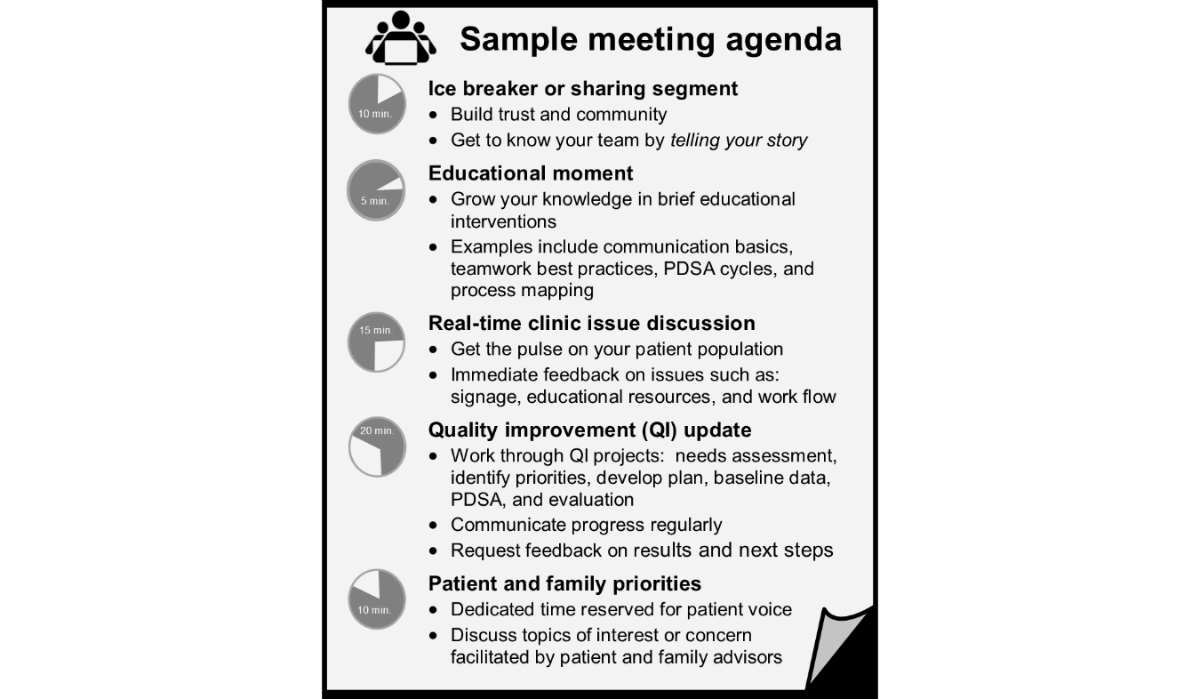
Patient and family advisory council sample meeting agenda. PDSA: Plan-Do-Study-Act.

### Patient and Family Advisory Council Meeting Launch

The initial meeting of the PFAC took place in fall 2016 and focused on key concepts for PFAC members, including basic health care concepts and confidentiality and communication strategies. Threaded throughout subsequent meetings were team-building exercises to build a sense of community, opportunities to practice sharing important health care–related stories, and discussion of current and relevant clinic issues to elicit immediate feedback from advisors. The basic tenants of clinic-based quality improvement, including tools like Plan-Do-Study-Act (PDSA) cycles, were also introduced, as were occasions to examine the office space and to identify areas for improvement projects (Figure 2).

**Figure 3:**
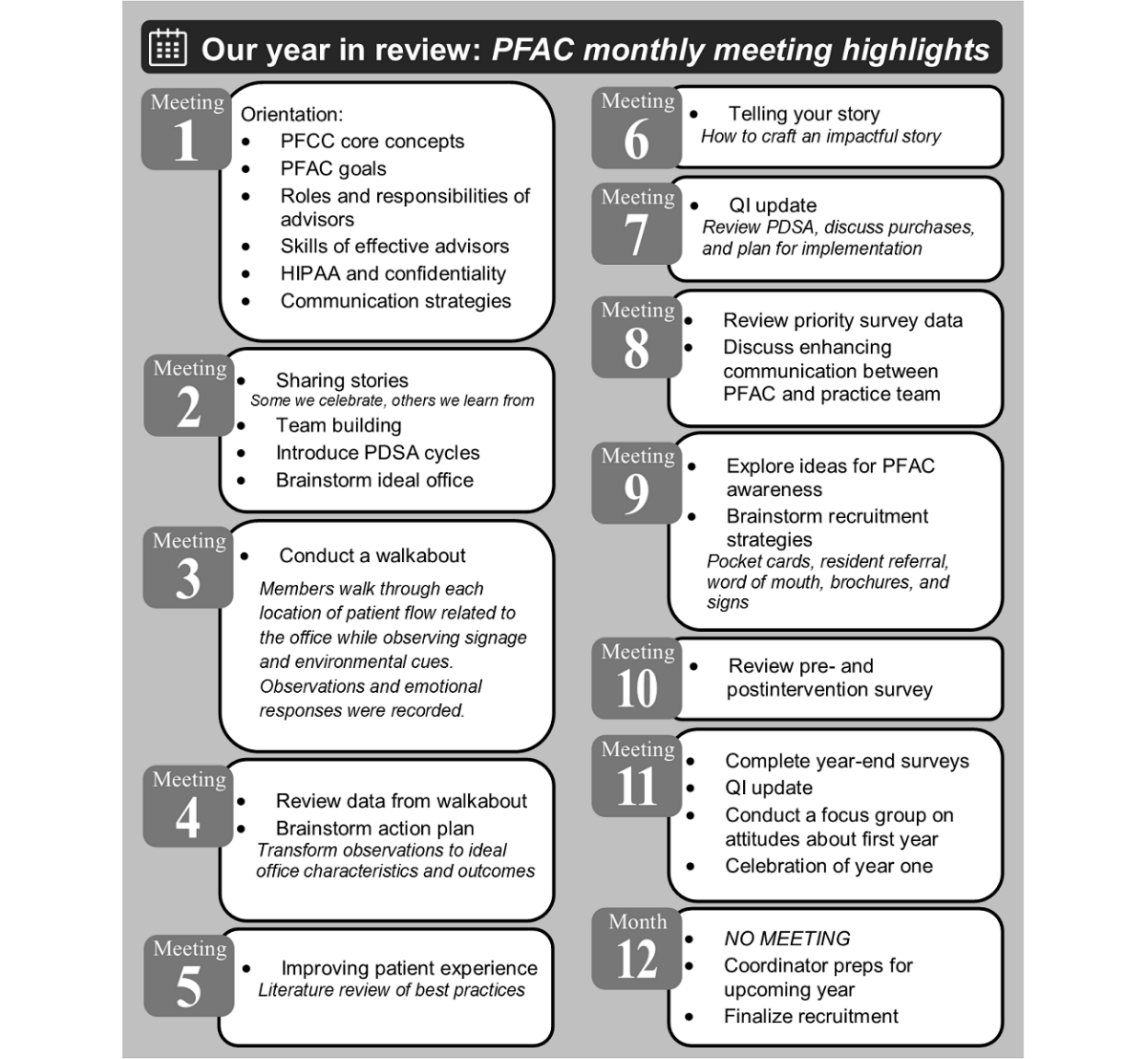
Patient and family advisory council monthly meeting highlights. HIPAA: Health Insurance Portability and Accountability Act; PDSA: Plan-Do-Study-Act; PFCC: patient- and family-centered care; QI: quality improvement.

PFAC recommendations helped identify the final projects: waiting room and exam room redesign to improve the patient experience. The final meetings focused on completing PDSA cycles including pre- and postintervention data collection and analysis (Figure 3).

### Measurement of Patient and Family Advisory Council Impact

To evaluate the PFAC implementation, we used a mixed-methods approach. We tracked the number of meetings held, number of attendees at each meeting, and roles of those who attended and conducted a focus group interview of the PFAC members at the end of the year to evaluate impressions, lessons learned, and suggestions for subsequent years. All PFAC members also completed an open-ended survey so that they could evaluate the year anonymously [9]. The evaluation team, consisting of a qualitative social science researcher, family physician, project manager, and premed student, analyzed data from open-ended surveys and focus group interviews, generating descriptive codes to create consensus themes.

## Results

### Group Composition

The composition of the PFAC consisted of 18 advisors, including 8 patient/family advisors, 4 staff advisors, 4 resident advisors, and 2 faculty advisors. The average meeting attendance was 12 members over 11 meetings in the span of the first year, with the most attended meeting having 15 members and the least attended having 9 PFAC members in attendance. Key leadership was present at every meeting, including the medical director and office nurse manager. Despite our best efforts, there was some attrition of patient and family advisors. By year’s end, there were 4 patient/family advisors regularly attending meetings, down from our 8 initial recruits.

### Qualitative Results

The PFAC’s first year concluded with feedback from the participants focusing on what they considered favorite aspects or experiences, impressions on their impact on the family medicine center, and guidance on future projects to improve patient care (Table 1). There were 13 respondents, including 7 patient and family advisors, 2 staff advisors, and 4 resident/physician advisors.

The topics identified common areas of the PFAC that members found to be positive and influential, indicating that it may be appropriate to continue to implement the corresponding experiences into the council’s regular operation. The walkabout was an important activity to experience the clinic through the patient lens and identify needs of the practice. Sharing stories is integral in identifying blind spots and providing context to know what needs to be improved. The PFAC involvement in practice meetings helped emphasize the patient and family voice and validate this partnership. The PFAC’s positive impact on the family medicine center was a result of building a vehicle for collaboration to improve the patient experience. Overall, 13 out of 13 (100%) advisors that completed the surveys were satisfied with their experience serving on the PFAC, with 1 member commenting:

It’s been great to be a part of something that is helping make a tangible change.

A common theme throughout centered on the value of member participation. This placed a focal point on ways in which PFAC members, particularly the patient and family advisors, can take a leading role in generating new ideas for the council to address in further driving the PFAC forward for the coming years.

**Table 1:** Qualitative data from the patient and family advisory council group.

Topic and theme		Quote
**Favorite aspects or experiences**		
	Walkabout	*That was the first time I had ever spent a significant amount of time in our waiting room, where it was before and just sitting down in one of those chairs it was like, Whoa, this gives me some perspective.* [Staff PFAC^a^ member]
		*I really enjoyed the walkabout and the conversations that followed. It felt like a really productive use of time and energy.* [Patient PFAC member]
	Sharing your story	*Sharing personal moments/stories at start of meeting helped understand where each member's perspective is built from. Also bond as a group*. [Patient PFAC member]
		*...sharing stories moment, in a safe place, talk about what it was like for my sister to just have walked through this health crisis and the thing that hurt the most was the thing the Medical Assistant said off-hand rooming her into the office. Just talk through these sorts of things.* [Resident PFAC member]
	PFAC involvement in practice meeting	*It was powerful to take PFAC concerns back to the FMC meeting and have that steer our efforts in QI development. The collective knowledge and goals from the group provide focus and force to getting projects accomplished, much more than any individual effort or goals could.* [Physician PFAC member]
	PFAC’s most positive impact on the family medicine center	*The PFAC's very existence and its positive reception by the med professional caregivers are the sources of any impact on care delivered at the Fam. Practice Center.* [Patient PFAC member]
		*Developing open communication lines between providers and patients.* [Patient PFAC member]
		*Incorporating patient voices into the development of new projects. Working to make it a more patient-centered culture.* [Patient PFAC member]
**Future projects to improve patient care: communication**		
	Phone	*I feel like something that is frustrating for me at times is when I call and have a question, whether it’s for a doctor or a nurse, and I kind of feel like it goes into a black hole, and I don’t get a response or it takes weeks. I would just love to see that process streamlined or figured out.* [Patient PFAC member]
	MyChart	*I think there is a breakdown of communication between patients, doctors, phone staff, and MyChart.* [Patient PFAC member]
	Waiting time	*Perhaps have updates for patients who have been roomed for >15 minutes. An update on where the provider is on the schedule may be helpful!* [Patient PFAC member]

^a^PFAC: patient and family advisory council.

Developing a safe environment with a strong sense of community and trust was the foundation for advisor engagement, promoting crucial input for relevant quality improvement ideas and ownership of projects. In analyzing future projects suggested by PFAC advisors, communication is at the core of each theme, a cornerstone of PFCC.

## Discussion

### Lessons Learned to Guide the Patient and Family Advisory Council’s Future

Our approach to PFAC implementation started with clinic and residency program leadership and garnered support before involving office staff or resident learners. Although leadership approval is certainly an important step early in the process, a top-down approach led to some limitations. We found that very early in the process, resident physicians and office staff seemed less invested in the PFAC and did not always recognize how collaboration with patient and family advisors could enhance the quality of care. Once up and running, investment was high, and in future iterations, we plan to take an interprofessional approach to involve leadership, physicians, residents, staff, and patients/family members from initial conception. Having representation from all stakeholders during the creation phase may prove beneficial to determine shared goals and promote support. The literature supports councils seeking to attain buy-in early to promote success [10].

The attrition rate of patient and family advisors was disappointing but not unexpected. There are many barriers to our patients attending meetings, from transportation problems to jobs with evening shifts to childcare. We were able to address some issues (such as childcare) but not all. The literature suggests most PFACs find it problematic to establish a diverse membership [11]. Although demographics in our office reflect the city composition with 50% patients of color, our PFAC had only 2 non-white members. For the upcoming cycle, we are recruiting more patient and family advisors, with focused recruitment on more diverse patients and families.

The literature highlights the need for an administrative manager to help make the PFAC more valuable and productive [11]. To keep attendance high, the PFAC coordinator kept in close contact with patient and family advisors through email, text, and phone calls based on advisors’ preferred forms of communication. This included reminders about upcoming meetings, sharing of “homework” assignments, and troubleshooting any potential conflicts that could interfere with meeting attendance. The estimated time required for coordinator oversight, not including work related to the collection of metrics related to the PFAC evaluation, was on average 10 hours per month. Time fluctuated throughout the year depending on the needs of the PFAC. Recruitment, implementation of projects, and year-end evaluation required additional time and effort.

Finally, as a residency training site with 26 learners, physician faculty are exploring ways to seamlessly incorporate PFAC concepts into the general residency teaching curriculum. Our PFAC is not only an ideal driver in identifying quality improvement projects but also serves as a critical partner in resident training. We have focused on harnessing patient and family advisors as teachers by inviting them to share at resident conferences. In addition, we now have patient and family advisors attend our practice meetings to promote buy-in, strengthen communication, and enhance their role as teachers. Although 3 to 4 resident physician PFAC members were felt to be ideally representative, the PFAC leadership team has also chosen to rotate residents every year to give as many residents as possible the chance to experience the PFAC. Though it is not possible for all residents to sit on the PFAC, more exposure to the patient and family advisors and their stories and perspectives can assist in driving home key concepts around patient participation and collaboration.

### Conclusions

The health care industry now recognizes that partnering with patients and families is a crucial step in optimizing health. This trend marks an evolutionary shift from the paternalistic practices of the past toward a new model of health care partnership between providers, patients, and families. Our residency-based family medicine center launched a PFAC in the fall of 2016 to collaborate with patients and family members beyond the traditional patient encounter. Our initial successes and lessons learned have cemented the belief in the positive outcomes possible with meaningful partnerships with patients and families.

## Appendix

Multimedia Appendix 1 Video abstract.

## Abbreviations

CMS: Centers for Medicare and Medicaid Services
CPC+: Comprehensive Primary Care Initiative Plus
PDSA: Plan-Do-Study-Act
PFAC: patient and family advisory council
PFCC: patient- and family-centered care
